# Abdominal Tumor in a 14-Year-Old Adolescent: Imperforate Hymen, Resulting in Hematocolpos—A Case Report and Review of the Literature

**DOI:** 10.1155/2015/429740

**Published:** 2015-02-08

**Authors:** George Marios Makris, Doris Macchiella, Dennis Vaidakis, Charalampos Chrelias, Marco Johannes Battista, Charalampos Siristatidis

**Affiliations:** ^1^Department of Obstetrics & Gynecology, Medical School, Johannes Gutenberg University of Mainz, Langenbeck Straße 1, 55131 Mainz, Germany; ^2^3rd Department of Obstetrics and Gynecology, Medical School, University of Athens, “Attikon” Hospital, Rimini Street 1, Chaidari, 12462 Athens, Greece

## Abstract

*Background*. Abdominal masses in female adolescents are uncommon. A rare cause of this condition is hematocolpos due to imperforate hymen. *Case*. We present a case of an unusually massive asymptomatic abdominal bulk in a 14-year-old female patient, who sought for medical advice after unusual abdominal pain lasting for few weeks. The patient was otherwise asymptomatic, apart from an unusual dramatic expansion of her abdominal wall during the last month. We describe the surgical management and the follow-up of the patient. *Summary and Conclusion*. Clinicians should keep in mind that an imperforate hymen can cause abdominal growth due to hematocolpos and include it in the differential diagnosis of such a clinical entity in female adolescents. 2D ultrasound is usually efficient for the confirmation of the diagnosis of hematocolpos, but 3D ultrasound is more accurate. Wide excision should be undertaken, as an initial approach, to avoid recurrence.

## 1. Introduction

An emerging abdominal tumor in young females is a rare situation and requires a specific clinical and ultrasonographic approach. Common causes of a newly diagnosed abdominal mass in young females include cysts and solid tumors of various origins. In this context, hematocolpos is a rare entity that can cause such symptoms: it comprises the blood collection in the distal closed vagina and is usually diagnosed in young adolescents with no menstruation and cyclic abdominal pain. Its incidence is approximately one every 2000 young adolescents [1] and in 90% of cases is caused by an imperforate hymen.

Usual clinical signs include cyclical low abdominal pain, urinary retention, back pain, primary amenorrhea, and/or a quickly enlarging pelvic tumor. It may also affect neonatal age and can be manifested as fetal ascites or renal failure [2, 3], sometimes leading to variable degrees of hydroureter and/or hydronephrosis [4]. For its diagnosis, 2D sonography is the usually indicated imaging method of choice. 3D sonography and MRI are rarely suggested and used, although they both provide a better visualization and differentiation of the tissues and a safer distinction among other causes of hematocolpos, such as vaginal septum or partial agenesis. In addition, an endocrine profile of the patient is usually necessary [5, 6]. Surgical management is the treatment of choice, through incision or excision of the hymen, using cold knife, scissors, electrocoagulation, or laser. The recurrence rate remains low, occurring more often during minor surgical approaches, such as after a cruciate incision. Notably, a spontaneous rupture of an imperforate hymen is likely to precede any decision for surgical management [7]. Finally, further issues have to be weighted, such as the bleeding and the subsequent emotional stress of the young female after the procedure, along with the completion and keeping of legal documentation.

## 2. Case

**Figure 1:**
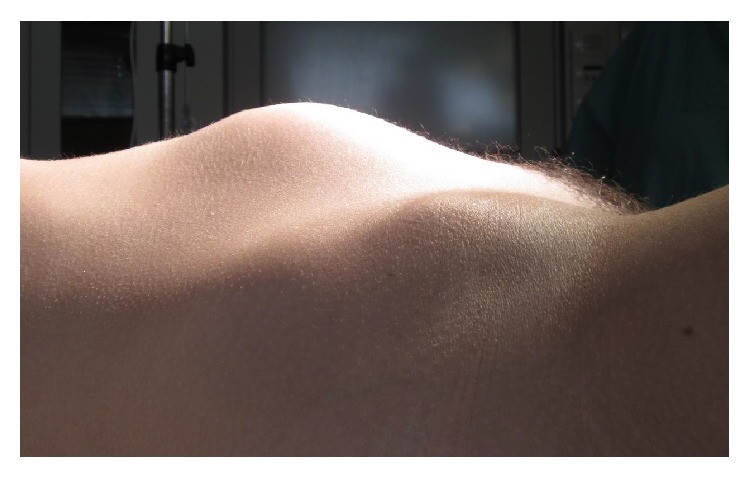
Clinical presentation of megahematocolpos.

A 14-year-old girl was admitted to the Pediatric Emergency Department of the Department of Obstetrics and Gynecology, Johannes Gutenberg University of Mainz, Mainz, Germany, with primary amenorrhea, an expanding abdominal mass, and mild abdominal pain. There was no history of severe abdominal pain during the last year and the patient complained of polyuria during the last month; there were no signs of defecation. Her parents sought for medical assistance because of a growing tumor in her abdomen. At clinical examination, secondary sexual characteristics were present and within normal ranges. The clinical presentation was quite impressive: a thin girl with a BMI of 22 with a painless, nontender, soft, and homogenous mass, distorting her abdominal wall and expanding up to 5 cm over the umbilicus (Figure 1).

**Figure 2:**
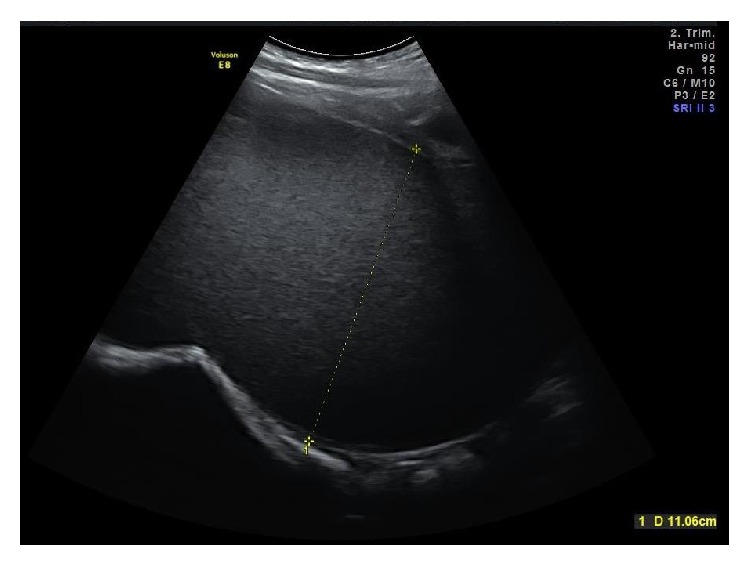
2D ultrasound imaging of megahematocolpos.

The patient's vital signs were normal; laboratory tests revealed a hemoglobin concentration of 13 g/dL and white blood cell count of 11/nL, while CRP and tumor markers' concentrations were within normal ranges. In addition, her endocrine hormonal profile was indicating a girl with a mature hypothalamic-pituitary axis. Urinalysis was normal. Clinical examination of the abdomen did not reveal any pain or signs of peritonism. Clinical gynecological examination after retracting the labia minor revealed an imperforate hymen, which was bulging forwards. Rectal digital examination revealed a large bulky mass positioned anteriorly. A structure of 34 cm length, 11 cm width, and 11 cm height was evidenced at 2D transabdominal ultrasound (Figure 2).

**Figure 3:**
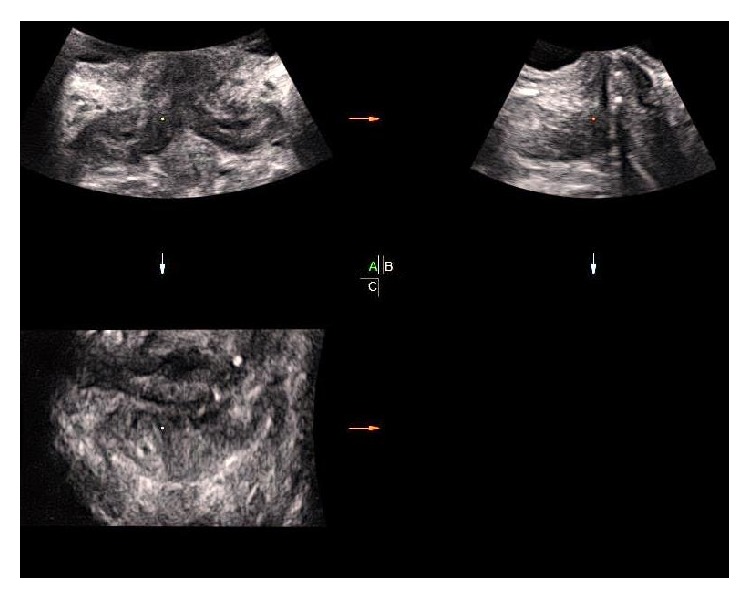
Postoperative 3D ultrasound imaging.

On the cranial, frontal end of the structure, cranial from the umbilicus and adapted to the front abdominal wall, we observed a uterus of normal size (no hematometra) (Figure 3), while both ovaries were present with a normal appearance. Both kidneys were present, with no anomalies or dilatation of the ureters.

3D ultrasound displayed the clarity of the wall of this structure: it appeared straight, with no adherence to the neighboring organs, homogenous, with a fluid-like content in it.

As the diagnosis was clear, surgical management was decided after providing the written informed consent of both parents and scheduled for the following day. A hymenotomy was performed under general anesthesia: at first, laser was used, followed by electrocoagulation, and an oval shaped piece of hymen was excised. A total sum of 2400 mL dark red, tarry blood was drained from the vagina. Of note, the maximum quantity reported in the literature is 3000 mL [8, 9]; spontaneous drainage was continued the following day too. No suturing of the remnant hymen was performed. Antibiotics were given prophylactically for the next 4 days.

3D imaging during the first postoperative day revealed a waveform vagina, with a length of approximately 21 cm, whereas the size of uterus regressed for 10 cm under the umbilicus but did not enter the minor pelvis. The patient was discharged from the hospital after two days and a weekly follow-up with 3D ultrasound was scheduled. Menstruation occurred 20 days postoperatively and vaginal length was normalized 3 days after. During a scheduled follow-up appointment, 2 months postoperatively, a small amount of blood was detected in the vagina through 3D imaging; recurrence of the hematocolpos was confirmed after genital inspection. Reoperation was booked immediately, leading to a wider triangular tissue excision.

The patient is not sexually active yet and during the last 12 months she has normal menstrual cycle and vaginal length, measured at ultrasound.

## 3. Summary and Conclusion

The approach of a young patient presenting with a newly diagnosed abdominal tumor is always a demanding process. It causes fear to the child, emotions of guilt to the parents, and additional responsibility to the clinician. Apart from the pediatrician, other medical specialties can assist towards diagnosis and management, such as general surgeon, gynecologist, endocrinologist, and radiologist.

Although hematocolpos consists of a rare clinical feature, it should be always considered a possible diagnosis in young females with primary amenorrhea and abdominal mass. Both diagnosis and treatment of hematocolpos are relative easy, but due to the sensitive nature of the disease, the approach of the patients presenting with that disease is demanding. Since the most serious complication is recurrence, in our experience, we recommend wide tissue excision as an initial approach, through a triangular or oval shape, instead of a cross or “X” shape incision. 2D ultrasound is the diagnostic tool of choice, but 3D ultrasound can reveal more details, such as the exact relationship of the feature with the neighboring organs and structures, since it provides better tissue differentiation and can assist in the vaginal length surveillance.
